# Enzyme engineering of cytochrome P450 RosC provides mechanistic insights into factors controlling iterative oxidation

**DOI:** 10.1007/s00253-025-13648-2

**Published:** 2025-12-09

**Authors:** Yohei Iizaka, Hironori Suzuki, Nanako Sasa, Kihika Ishiuchi, Yuta Kumakiri, Haruki Kawasaki, Hayato Sato, Kanon Fujimoto, Shuji Noguchi, Yojiro Anzai

**Affiliations:** Faculty of Pharmaceutical Sciences, Toho University, 2-2-1 Miyama, Funabashi, Chiba Japan

**Keywords:** Cytochrome P450 enzymes, Iterative oxidation, Multistep catalysis, Reaction control, Crystal structure, Macrolide biosynthesis

## Abstract

**Abstract:**

Cytochrome P450 enzymes capable of performing iterative oxidation at the same substrate site contribute to compound diversification; however, reaction control is also necessary for the efficient production of the desired compound. RosC, a cytochrome P450 enzyme involved in the biosynthesis of the 16-membered ring macrolide antibiotic rosamicin, catalyzes stepwise oxidation of the ethyl group at C-20 via hydroxylation to an alcohol, followed by successive oxidation to the corresponding aldehyde and carboxylic acid. The P107S/L176Q mutant produces the hydroxylated intermediate in the first oxidation step, but the efficiency of the subsequent conversion to aldehyde and carboxylic acid is significantly reduced. To elucidate the factors responsible for the reduced efficiency of the second and subsequent oxidation steps in the P107S/L176Q mutant and to understand how RosC facilitates multistep oxidative modification, we compared the reaction time courses and substrate-binding affinities of RosC and the P107S/L176Q mutant. The mutant exhibited a reduced reaction rate for the initial hydroxylation and showed reduced substrate-binding affinities for both the hydroxylated and aldehydic intermediates.

Furthermore, crystallographic analysis revealed that Leu-176 played a key role in binding to the desosamine moiety of the substrate, and its mutation resulted in the loss of this function. Ser-248 was presumed to play a role in re-anchoring the modification site to the active center through hydrogen bonding with the hydroxyl and aldehyde groups generated in the first and second reactions. These findings are expected to contribute to the multifunctionalization of cytochrome P450 enzymes and the regulation of their reactivity.

**Key points:**

• *RosC-catalyzed three-step oxidation is limited to one step in the P107S/L176Q mutant.*

• *P107S/L176Q mutations impair substrate binding by increasing structural fluctuations.*

• *Leu-176 and Ser-248 position the substrate for RosC-catalyzed iterative oxidation.*

**Graphical Abstract:**

**Figure:**
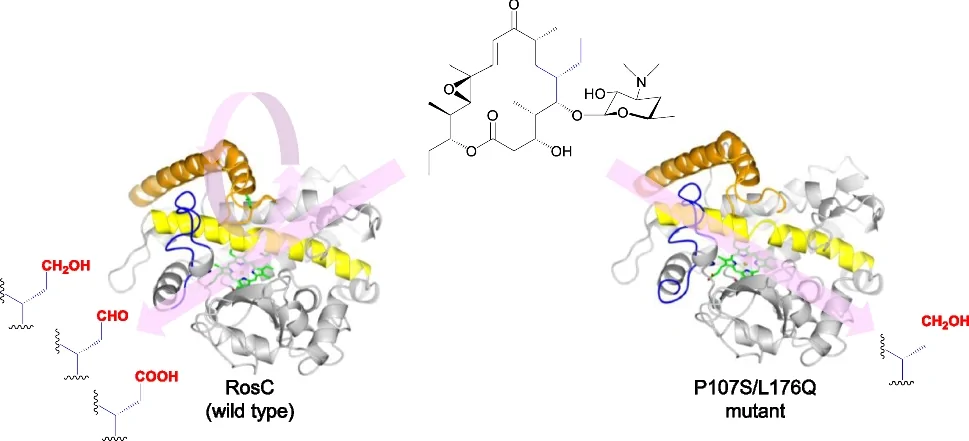

**Supplementary Information:**

The online version contains supplementary material available at 10.1007/s00253-025-13648-2.

## Introduction

Cytochrome P450 enzymes (P450s) are a family of heme-containing proteins found in a wide range of organisms, from bacteria and plants to mammals (Omura 2010; Nelson 2018). Most of the P450s identified thus far share a common dioxygen activation mechanism, wherein a single oxygen atom is inserted into the C–H bond of the target substrate, as well as a characteristic three-dimensional structure with a triangular prism-like fold (Meunier et al. 2004; Denisov et al. 2005; Guengerich 2007). Despite these common features, P450s catalyze a wide range of reactions, including hydroxylation, epoxidation, dealkylation, heteroatom oxidation, and C–C bond formation/cleavage, and play crucial roles in xenobiotic metabolism and endogenous biosynthesis (Sono et al. 1996; Isin & Guengerich 2007; Guengerich & Munro 2013).

Actinomycetes produce a broad array of biologically active secondary metabolites that exhibit antibacterial, antifungal, antitumor, and immunosuppressive properties (Jose et al. 2021). Various P450s are involved in the biosynthesis of these secondary metabolites, contributing to their structural diversity and bioactivity (Rudolf et al. 2017; Greule et al. 2018; Cho et al. 2019). These enzymes have been used in biocatalytic and chemoenzymatic syntheses for the production of useful or unnatural compounds, as they catalyze regio- and stereoselective reactions that often involve intricate processes in organic synthesis (Li et al. 2020; Tanifuji & Oguri 2024). While P450s that catalyze a single oxidation reaction are more common in the biosynthesis of secondary metabolites, there are some P450s that catalyze multiple oxidation reactions and have been characterized as iterative or multifunctional P450s (Cochrane and Vederas 2014; Iizaka et al. 2021). The substrates recognized by these P450s show no consistent structural features, and the enzymes are distributed across various CYP families. The utilization of P450s, which catalyze multiple oxidation reactions, as biocatalysts provides a significant advantage because a single enzyme can modify the structure of a compound multiple times and simultaneously induce changes in its biological activity. However, if a target compound produced using these P450s is an intermediate in a multistep reaction, consecutive oxidation reactions may convert it into an unintended product, thereby reducing its production efficiency. Therefore, understanding the reaction mechanisms and regulatory processes of these enzymes is essential for ensuring their efficient use.

**Fig. 1:**
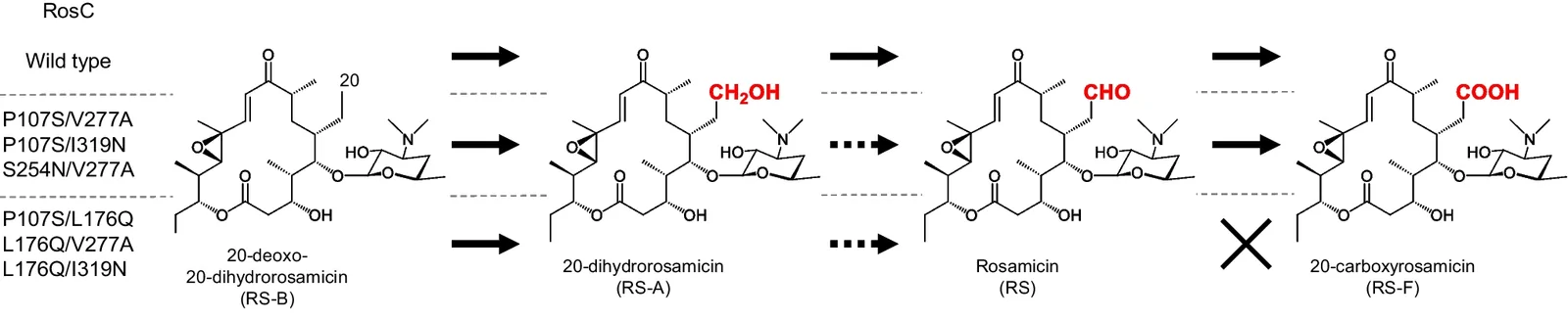
Alteration of multistep oxidation reactions catalyzed by RosC and its mutants. Dashed arrows represent reactions with low conversion efficiency in whole-cell assays using *E. coli*, as demonstrated in previous studies (Iizaka et al. 2020)

P450s RosC and RosD, belonging to the CYP113B and CYP107 families, respectively, are responsible for the final step in the biosynthesis of rosamicin (RS), a 16-membered ring macrolide antibiotic produced by *Micromonospora rosaria* IFO 13697 (Iizaka et al. 2013, 2017). This step involves a branched oxidation pathway. RosC catalyzes a three-step oxidative process at C-20 that comprises hydroxylation, alcohol oxidation, and aldehyde oxidation. RosD plays a role in epoxidation of the macrolactone ring at C-12/13. In our previous study, we generated RosC mutants with altered catalytic activity for multistep oxidation reactions by combining random mutagenesis with subsequent site-specific mutations (Fig. 1) (Iizaka et al. 2020). The P107S/L176Q, P107S/V277A, P107S/I319N, L176Q/V277A, L176Q/I319N, and S254N/V277A mutants significantly reduced the efficiency of the second reaction, which converts the alcohol moiety to an aldehyde, in whole-cell assays using *Escherichia coli* expressing RosC mutants. Among these mutants, double mutants containing L176Q lacked the ability to convert the aldehyde moiety of RS to a carboxylic acid in the third reaction. Additionally, a tenfold increase in the production of the hydroxylated RS biosynthetic intermediate was observed in the engineered *M. rosaria* strain reconstituted with the P107S/L176Q mutant, and almost no aldehydic or carboxylated forms were detected compared with those in the wild-type strain. However, the precise mechanism whereby these mutations alter the three-step oxidation reaction catalyzed by RosC remains unclear. In this study, we aimed to elucidate how RosC achieves site-specific multistep oxidation and how this process is regulated by amino acid substitutions. To this end, we compared the time course of the in vitro reaction, substrate-binding affinities, and X-ray crystal structures of RosC and the P107S/L176Q mutant.

## Materials and methods

### Expression and purification of RosC-related proteins (RosCs)

The genes encoding RosC and the RosC_P107S/L176Q_ mutant were amplified using PCR with the primers rosC_Nd-F/rosC_Xh-R (Table S1; Genbank accession number AB740326). After digestion of the PCR products with *Nde*I and *Xho*I, they were inserted into the corresponding sites of the pET28b vector (Novagen, WI, USA). Each gene encoding RosC or the RosC_P107S/L176Q_ mutant with deletions of the N-terminal 21 residues was inserted into pET28b in a similar manner using the primers shown in Table S1. *E. coli* BL21 (DE3) (Stratagene, CA, USA) transformed with each expression plasmid was cultivated at 32 °C for 20 h in Luria–Bertani medium (0.5% yeast extract, 1.0% tryptone, and 1.0% sodium chloride [NaCl], pH 7.2) containing kanamycin (50 μg/mL). The seed culture (10 mL) was transferred into 1 L of Luria–Bertani medium containing kanamycin (50 μg/mL), and cultivated at 37 °C. When the optical density at 600 nm reached a value of 0.8, isopropyl-β-D-thiogalactoside and 5-aminolevulinic acid were added to a final concentration of 0.1 mM and 80 μg/mL, respectively. After further growth for 20 h at 22 °C, the cells were harvested through centrifugation and frozen at − 80 °C.

After thawing under running water, cells were suspended in 100 mL of buffer A (50 mM Tris–HCl [pH 7.5], 500 mM NaCl, and 0.2 mM dithiothreitol) containing 0.5 mg/mL of lysozyme and 6.25 unit/mL of benzonase. The cell suspension was sonicated using an ultrasonic disruptor UD-200 (TOMY, Tokyo, Japan) and centrifuged to remove cell debris. The supernatant was loaded onto a nickel-nitrilotriacetic acid-agarose (Ni–NTA) column preequilibrated with buffer A. Nonspecifically bound proteins were removed by washing with buffer B (50 mM Tris–HCl [pH 7.5], 100 mM NaCl, and 0.2 mM dithiothreitol). The column was first washed with buffer B containing 5 mM imidazole, followed by washing with buffer B containing 40 mM imidazole. N-terminally His-tagged (NHis) RosCs were eluted with buffer B containing 250 mM imidazole. The purified proteins were concentrated using 30 kDa MWCO centrifugal filters, and desalting and concentration were performed with repeated addition of buffer B. For biochemical experiments, the purified NHis-RosCs were flash-frozen in liquid N_2_ and stored at − 80 °C.

To obtain RosCs for X-ray crystallographic analysis, 5 mg of NHis-RosCs were allowed to react with 40 units of biotinylated thrombin for 4 h at 25 °C. Streptavidin agarose was added to the reaction mixture, which was then subjected to a spin column to remove thrombin. The collected filtrate was passed through a Ni–NTA column pre-equilibrated with buffer B to remove the His-tag fragments. The passing fraction containing RosCs was further purified using an Äkta start system (GE Healthcare, Chicago, IL, USA) with a 5-mL HiTrap Q HP anion exchange column (GE Healthcare, Chicago, IL, USA) that was previously equilibrated with buffer C (20 mM Tris–HCl [pH 7.5], and 200 mM NaCl) at a flow rate of 2 mL/min. After washing for 5 min, the samples were eluted with a linear NaCl concentration gradient ranging from 200 to 1000 mM for 30 min. The fractions containing RosCs were concentrated using a 30 kDa MWCO centrifugal filter, and buffer D (20 mM Tris–HCl [pH 7.5], 150 mM NaCl) was added to adjust the salt concentration. Samples were applied to a HiLoad 16/600 Superdex 200 pg column (GE Healthcare, Chicago, IL, USA) under the control of an Äkta start system using the mobile phase buffer D at a flow rate of 1 mL/min. The 135-mL portion was fractionated 5 mL at a time, and the fractions containing RosCs were pooled and concentrated. Then, 20 mg/mL of purified RosCs was flash frozen in liquid N_2_ and stored at − 80 °C until use for crystallization. Protein purity at each purification step was confirmed using sodium dodecyl sulfate–polyacrylamide gel electrophoresis. The concentration of correctly folded RosCs was calculated from the CO difference spectra using the extinction coefficient of 91 mM^−1^ cm^−1^ at 450 nm (Guengerich et al. 2009).

### Crystallization

Since high-quality crystals could not be obtained from the full-length proteins, constructs of RosC and RosC_P107S/L176Q_ lacking the N-terminal 21 residues were used for crystallization. Crystals of RosC and RosC_P107S/L176Q_ were grown at 20 °C using the hanging-drop vapor-diffusion method from an equivolume mixture of 10 mg/mL proteins in buffer D and a reservoir solution consisting of 24% PEG3350 and 0.1 M Bis–Tris pH 5.50 − 5.75. The substrate-saturated solution was prepared by adding 0.5 mM 20-deoxo-20-dihydrorosamicin (RS-B), 20-dihydrorosamicin (RS-A), and RS to 10 mg/mL of RosC solution just before crystallization. Crystals of RosC in the presence of its substrates were grown at 20 °C using the sitting-drop vapor-diffusion method from an equivolume mixture of 10 mg/mL proteins in buffer D containing 0.5 mM each substrate at a molar ratio of 1:2 and a reservoir solution consisting of 60% Tacsimate (pH 6.0). The crystallization conditions of RosC, RosC_P107S/L176Q_, and RosC complexed with substrates were further optimized for diffraction data collection (Table S2).

### Data collection and structure determination

The diffraction datasets were collected at beamlines BL-5A, BL-17A, and AR-NE3A at the Photon Factory (Tsukuba, Japan). Prior to data collection, the crystals of RosC and RosC_P107S/L176Q_ were soaked for a few seconds in the reservoir solution supplemented with 10 − 20% ethylene glycol and flash-cooled to − 178 °C. In the case of the crystals of RosC complexed with its substrate, the corresponding substrates (250 μM) were also added to the reservoir solution and flash-cooled in the same manner. The diffraction data were indexed, integrated, and scaled using XDS (Kabsch 2010). The initial phase of RosC was determined using the molecular replacement method using the MOLREP program implemented in the CCP4 suite with the CYP109E1 structure (PDB ID: 5I90) as the search model (Collaborative Computational Project, Number 4, 1994). The initial phases of RosC_P107S/L176Q_ and RosC complexed with its substrates were determined using the molecular replacement method, using the refined RosC structure as a search model and the MOLREP program implemented in the CCP4 package. Model building and adjustment were performed using COOT software (Emsley et al. 2010). Crystallographic refinement was performed using PHENIX until the *R* factor converged (Table S2) (Liebschner et al. 2019). Atomic coordinates and structural factors have been deposited in the Protein Data Bank (PDB codes 9VGM, 9VGN, 9VGQ, 9VGP, and 9VGO for RosC, RosC_P107S/L176Q_, RosC complexed with RS-B, RosC complexed with RS-A, RosC complexed with RS, respectively).

### Functional analysis of in vitro activities of RosC and RosC_P107S/L176Q_

RS used as the substrate for the enzymatic reaction was purchased from Sigma-Aldrich Japan. RS-B and RS-A were isolated and purified from the fermentation broth of the *rosC* disruption strain and the RosC_P107S/L176Q_-expressing strain of *M. rosaria*, respectively, as described previously (Iizaka et al. 2013, 2020). Enzymatic reactions were performed under the following conditions: 25 mM Tris–acetate (pH 7.5), 10% (v/v) glycerol, 1 mM NADPH, 0.1 mg/mL spinach ferredoxin, 0.1 U/mL spinach ferredoxin-NADP^+^ reductase, 5 mM glucose-6-phosphate, 1 U/mL glucose-6-phosphate dehydrogenase, 100 μM RS biosynthetic intermediates, and 1 μM NHis-RosC or NHis-RosC_P107S/L176Q_. The total volume of each reaction was 200 μL, and reaction mixtures were incubated at 27 °C for 20 h. The reactions were terminated by the addition of 20 μL of acetic acid. After the reaction mixture was centrifuged at 2,000 × *g* for 5 min, 20 μL of the supernatant was subjected to high-performance liquid chromatography (HPLC) analysis. HPLC runs were performed on a Hitachi Chromaster HPLC system (Hitachi High-Tech, Tokyo, Japan) with a TSKgel ODS-80TM column (4.6 mm i.d. × 150 mm; Tosoh, Tokyo, Japan), using the mobile phase acetonitrile/0.06% trifluoroacetic acid (35:65) (flow rate, 0.8 mL/min). Each RS derivative was quantified on the basis of its HPLC peak area, which was calibrated using the peak area of a standard solution.

Time-course analysis between NHis-RosC or NHis-RosC_P107S/L176Q_ and RS-B was performed under the same conditions as described above, provided that the reactions were initiated by the addition of NAPDH and incubated for 0, 5, 30, 60, 120, 180, 360, and 540 min. All reactions were performed in duplicates and reproducibility was confirmed.

### Spectral substrate-binding assay

Spectroscopic substrate-binding assays were performed using a Gene Quant 1300 UV–Vis spectrophotometer (GE Healthcare, Chicago, IL, USA) at room temperature. Spectra were obtained by diluting NHis-RosC or NHis-RosC_P107S/L176Q_ in 50 mM Tris–HCl (pH 7.5), containing 20% (v/v) glycerol, to obtain a 1 μM solution and then adding the substrate dissolved in dimethyl sulfoxide at the suitable range of concentrations. The condition with no substrate added was used as a baseline. Average absorbance differences ΔA (A_peak_-A_trough_) from experiments performed in duplicate were plotted versus substrate concentration. The dissociation constant *K*_d_ was calculated by a nonlinear regression fit to the Michaelis–Menten equation using SigmaPlot 15.

## Results

### Catalytic activities of RosC and RosC_P107S/L176Q_

**Fig. 2:**
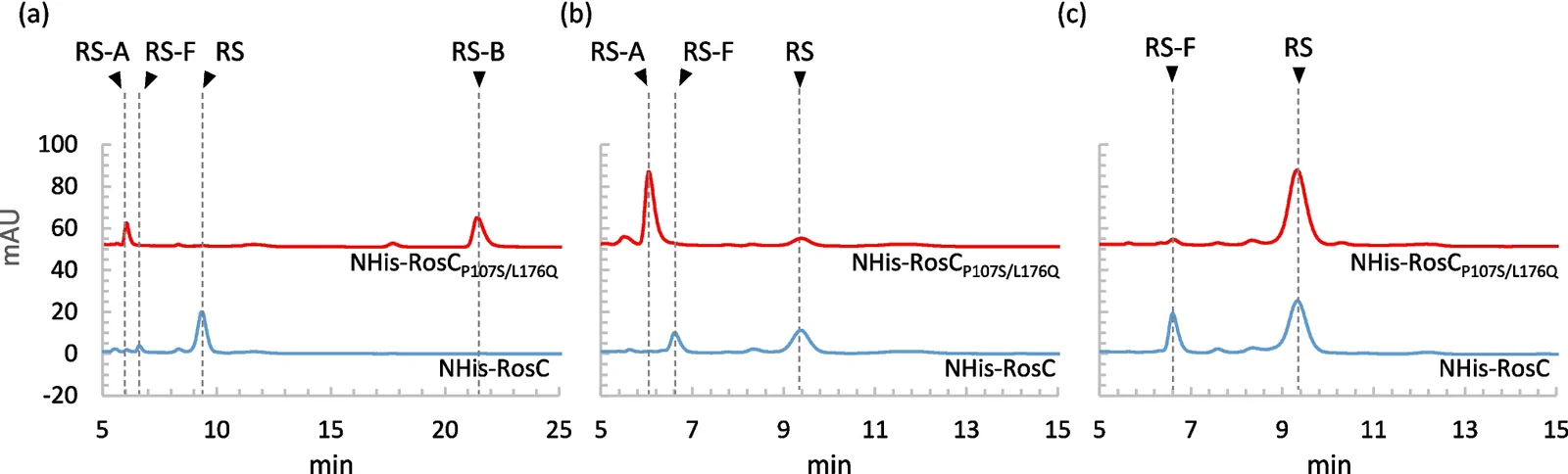
HPLC analysis (240 nm) of the enzymatic reactions catalyzed by NHis-RosC and NHis-RosC_P107S/L176Q_ using RS-B (**a**), RS-A (**b**), and RS (**c**) as substrates

P450s typically require redox partners to exert their catalytic activity. However, as with many P450s involved in natural product biosynthesis, the intrinsic redox system of RosC has not been elucidated. In this study, spinach-derived ferredoxin and ferredoxin-NADP^+^ reductase, widely employed in P450 characterization, were utilized as the redox system (Anzai et al 2008; Li et al. 2009). To evaluate the enzymatic activities of RosC and RosC_P107S/L176Q,_ NHis-RosC and NHis-RosC_P107S/L176Q_ were incubated with RS-B, RS-A, and RS in vitro for 20 h (Fig. 2 and Table 1). RosC generated RS-A, RS, and 20-carboxyrosamicin (RS-F) from substrate RS-B through two- and three-step oxidation reactions with conversion rates of 3.6%, 85.2%, and 10.9%, respectively. In contrast, RosC_P107S/L176Q_ converted 35.7% of RS-A formed in a single-step oxidation reaction without generating RS or RS-F. When RS-A was used as the substrate, RosC converted 66.3% of it to RS and 33.7% to RS-F, whereas RosC_P107S/L176Q_ converted 10.4% of it to RS. The efficiency of RS-to-RS-F conversion was also significantly lower with RosC_P107S/L176Q_ than with RosC. The finding that RosC_P107S/L176Q_ exhibited significantly lower catalytic activity in the second and subsequent steps of the oxidation reaction was generally consistent with the results of whole-cell assays using *E. coli* shown in our previous study (Iizaka et al. 2020).

**Table 1:** Relative contents of RSs in the reaction mixtures of NHis-RosC and NHis-RosC_P107S/L176Q_

RosC	Substrates	Relative contents (%)			
		RS-B	RS-A	RS	RS-F
NHis-RosC	RS-B	0.3 ± 0.1	3.6 ± 0.0	85.2 ± 2.7	10.9 ± 2.8
NHis-RosC_P107S/L176Q_	RS-B	64.3 ± 0.7	35.7 ± 0.7	0.0	0.0
NHis-RosC	RS-A	-	0.0	66.3 ± 4.1	33.7 ± 4.1
NHis-RosC_P107S/L176Q_	RS-A	-	89.6 ± 1.3	10.4 ± 1.3	0.0
NHis-RosC	RS	-	-	57.7 ± 4.3	42.3 ± 4.3
NHis-RosC_P107S/L176Q_	RS	-	-	93.4 ± 0.6	6.6 ± 0.6

The average percentages of relative contents and their standard deviations reflect the results from duplicate experiments

**Fig. 3:**
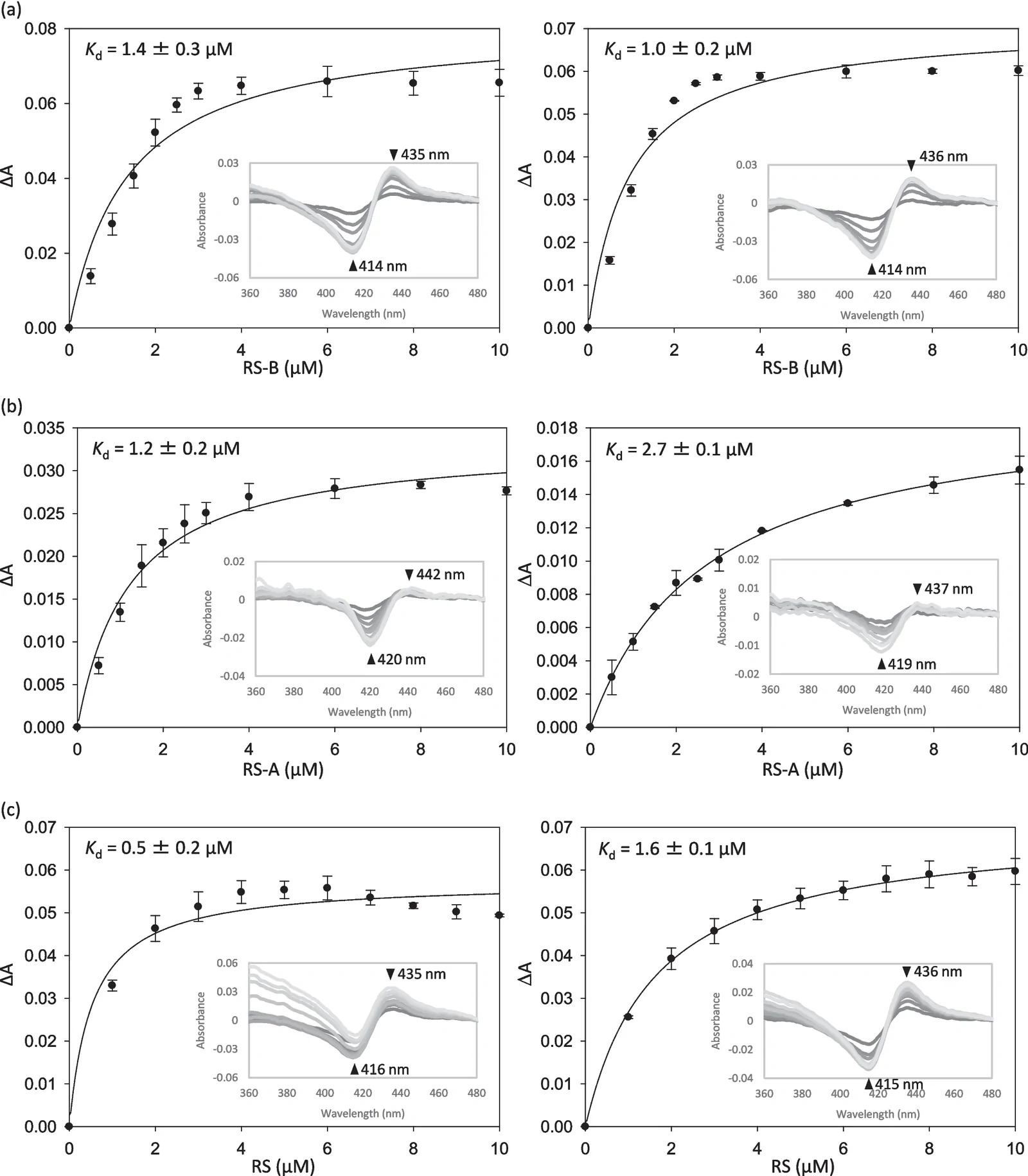
Measurement of the substrate dissociation constants of RS-B (**a**), RS-A (**b**), and RS (**c**) for NHis-RosC (left) and NHis-RosC_P107S/L176Q_ (right). The inset shows the difference spectra recorded at each substrate concentration. The differences in absorbance between the peak and trough of the difference spectra were plotted against the substrate concentration, and the resulting data were fitted by nonlinear regression using a one-site specific binding equation to estimate the *K*_d_. Data represent the average of values from two independent experiments, and error bars indicate the standard deviation of mean

Then, we performed spectrophotometric substrate-binding assays to determine the dissociation constants (*K*_d_) of NHis-RosC and NHis-RosC_P107S/L176Q_ for each substrate to understand the differences in conversion efficiency described above. Intriguingly, RosC exhibited Type II spectral changes with a positive peak at 436 nm and a negative trough at 416 nm upon substrate binding (Fig. S1) (Luthra et al. 2011). P450 RosD, which catalyzes epoxidation at C-12/13 in RS biosynthesis (Iizaka et al. 2013), showed typical Type I spectral changes. Generally, many P450s exhibit Type I spectral changes by displacing the heme water ligand with a substrate and shifting the iron spin equilibrium toward a high-spin state. Type II spectral changes occur when the water ligand is replaced by a nitrogen-containing heterocyclic ring or an aniline group with a higher affinity, thereby stabilizing the low-spin state (Locuson et al. 2007; Podgorski et al. 2022). Although the mechanism whereby RosC exhibits Type II spectral changes upon binding to RSs remains unclear, substrate concentration-dependent spectral changes were observed, facilitating the evaluation of dissociation constants using a spectral binding assay (Fig. 3). RS-B bound to RosC and RosC_P107S/L176Q_, with *K*_d_ values of 1.4 ± 0.3 and 1.0 ± 0.2 μM, respectively, indicating comparable binding affinities. In contrast, RS-A and RS exhibited approximately 2- to threefold lower binding affinities for RosC_P107S/L176Q_ than for RosC. These trends were consistent with the reduced conversion efficiency of RosC_P107S/L176Q_ when RS-A and RS served as substrates in the enzymatic reactions described above. Nevertheless, the *K*_d_ values of RosC_P107S/L176Q_ for the individual RSs were sufficiently low to preclude definitive identification of the factor responsible for the lower conversion efficiency.

**Fig. 4:**
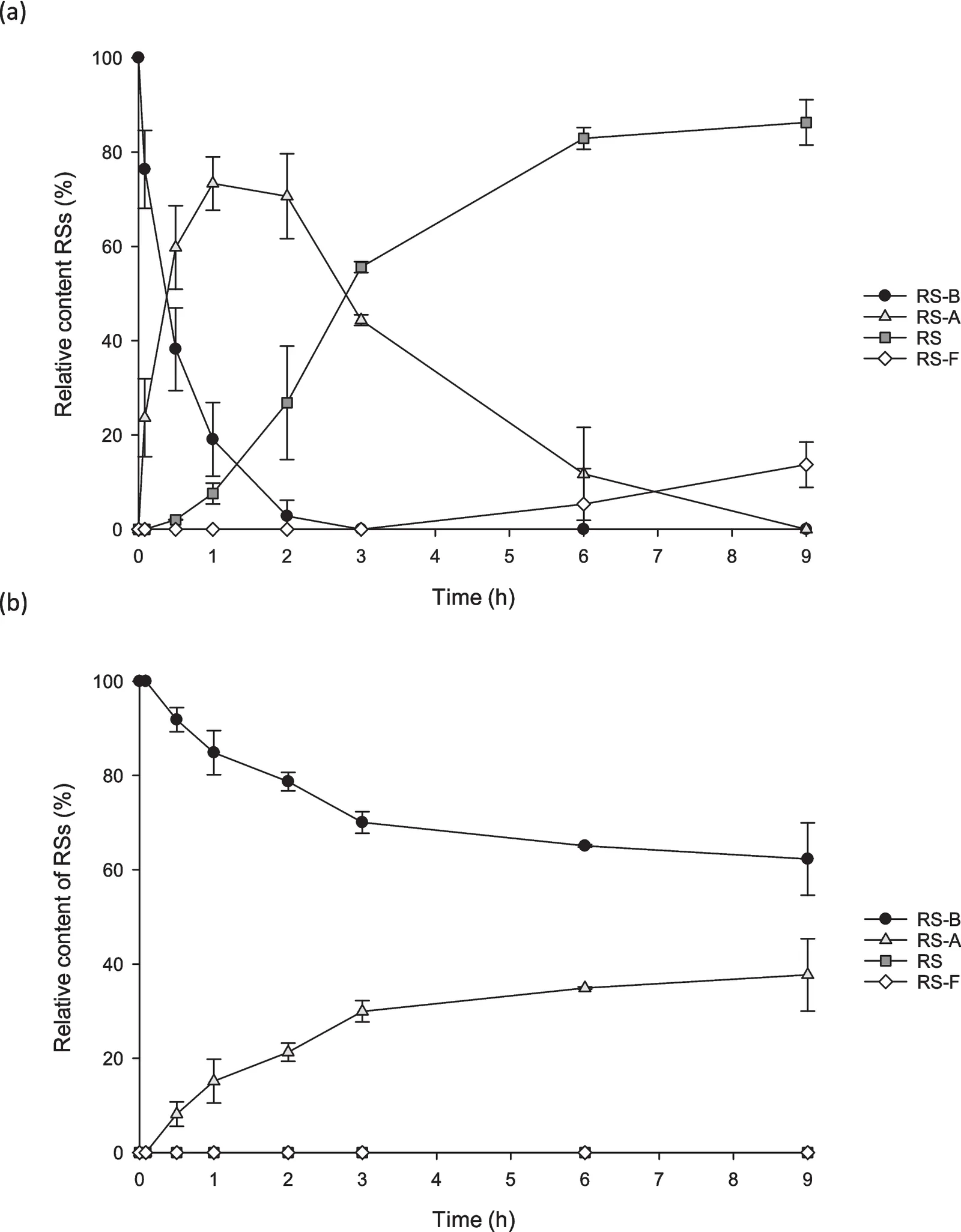
Time course of the enzymatic reactions catalyzed by NHis-RosC (**a**) and NHis-RosC_P107S/L176Q_ (**b**), with RS-B as the substrate. Data represent the average of values from two independent experiments, and error bars indicate the standard deviation of mean

Given the challenges of performing kinetic analysis, as RosC catalyzes a multistep reaction, we analyzed the time course of the reaction of NHis-RosC and NHis-RosC_P107S/L176Q_ with RS-B to understand why the variant catalyzed only a single-step conversion from RS-B to RS-A (Fig. 4 and Table S3). RosC converted 23.7% of RS-B to RS-A within 5 min, and 73.3% of RS-A and 7.6% of RS were detected within 1 h. Subsequently, RS formation increased, and RS-F was observed after 6 h (Fig. 4a). RosC_P107S/L176Q_ converted 8.2% of RS-B to RS-A within 30 min, and then increased RS-A to 28.8% within 3 h (Fig. 4b). RS-A continued to increase slightly for up to 9 h, with neither RS nor RS-F being generated. These results indicated that the catalytic efficiency of RosC_P107S/L176Q_ in generating RS-A from RS-B was significantly reduced. The fact that RosC_P107S/L176Q_ produces only RS-A from RS-B is largely attributed not only to its reduced binding affinity for RS-A and RS but also to the significantly decreased catalytic efficiency of the first-step oxidation.

### Crystal structure of ligand-free RosC and RosC_P107S/L176Q_

**Fig. 5:**
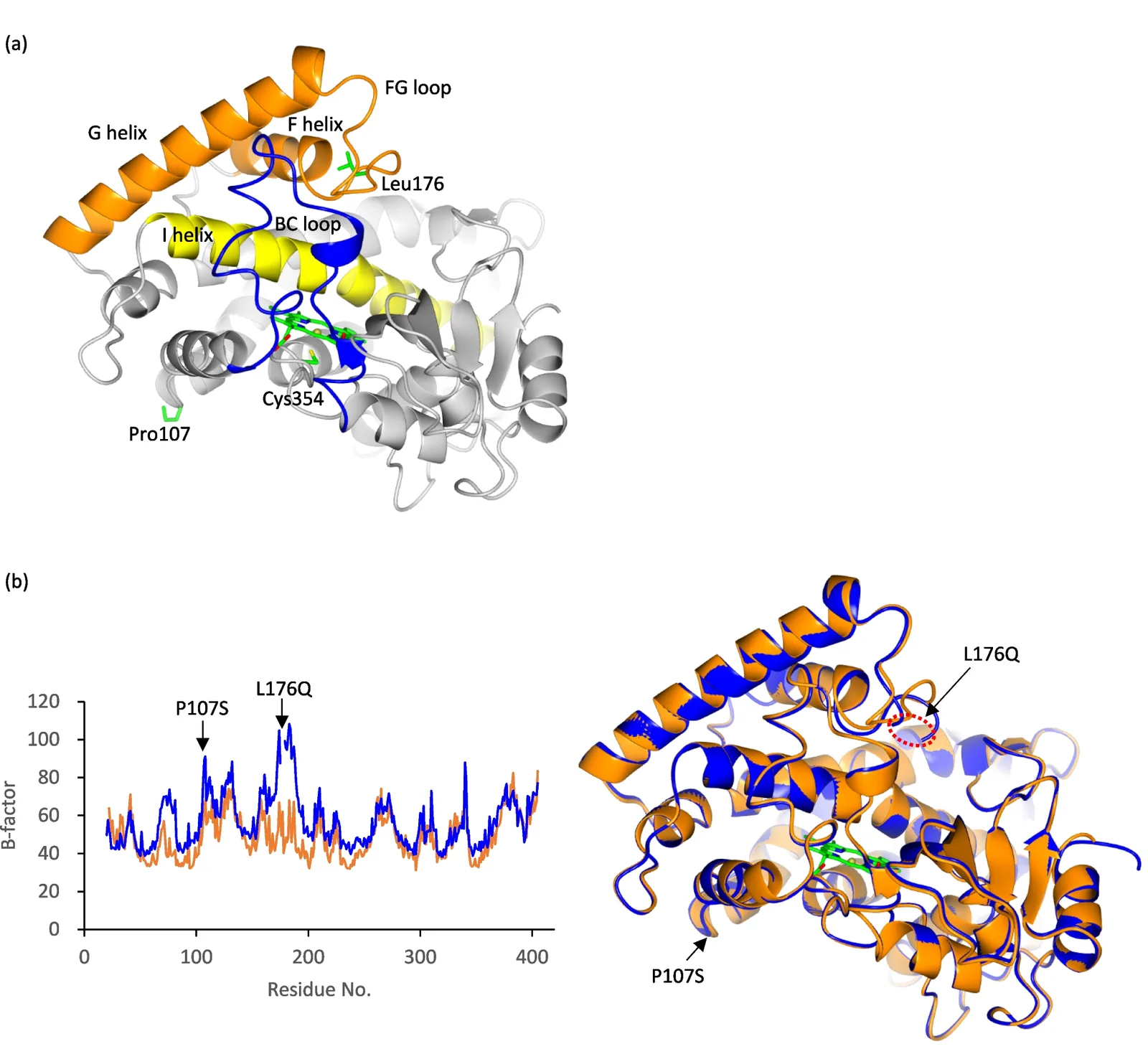
Structures of RosC and comparison with RosC_P107S/L176Q_. (**a**) Overall structures of RosC. Pro-107, Leu-176, Cys-354, and heme are shown as *stick models*. The BC loop region, FG helix region, and I helix region are shown in blue, orange, and yellow, respectively. (**b**) Comparison of B-factors and superimposed structures of RosC (*orange*) and RosC_P107S/L176Q_ (*blue*). The mutation sites of P107S and L176Q are denoted by *arrows*. The red dotted box indicates a region spanning Glu-174 to Tyr-178 of RosC_P107S/L176Q_ where the model structure could not be built

As the N-terminus of RosC was predicted to be flexible based on secondary structure prediction using PSIPRED analysis, both RosC and RosC_P107S/L176Q_ were prepared in full-length form and in an N-terminally truncated form lacking 21 residues (McGuffin LJ et al. 2000). Consequently, crystals of both proteins were successfully obtained using the truncated constructs. The crystal structure of RosC was determined at 2.15 Å resolution (Table S2). Three monomers—A, B, and C—are present in the asymmetric unit of the *C*222_1_ space group. The crystal structure of RosC_P107S/L176Q_ was determined at 2.45 Å resolution, which is isomorphous to the RosC. Three monomers in each structure superimpose well with root mean square deviations (rmsd) of the main chain atoms ranging from 0.22 to 0.29 Å for the RosC and 0.37 to 0.38 Å for the RosC_P107S/L176Q_ (Table S4). The structures of RosC and RosC _P107S/L176Q_ were also superimposed, with rms positional differences of the main chain atoms ranging from 0.29 to 0.49 Å. Therefore, we will focus on only monomer A out of the three monomers in the following discussion. RosC adopts a typical triangular P450 fold, in which Cys-354 acts as the fifth thiolate ligand coordinating with the heme iron, and a long I helix runs through the entire molecule above the sixth coordination site (Fig. 5a). The BC and FG loops partially cover the I-helix. A comparison of the crystal structures of RosC and RosC_P107S/L176Q_ revealed that the mutations led to increased B-factor values and greater structural fluctuations, although the overall structures were nearly identical (Fig. 5b). In particular, in the crystal structure of RosC_P107S/L176Q_, residues 174 − 178 of monomer A, 175 − 179 of monomer B, and 175 − 177 of monomer C, which were located in the FG loop, were not visible in the electron density map; therefore, these residues were not included in the model (Fig. S2). This region was assumed to be disordered even within the crystal. The Pro-107 to Ser mutation on the C-helix was accompanied by increased B-factors, and residues 68–81 in the BC loop also showed elevated B-factors. The latter increase is presumed to be associated with both the C-helix mutation at the base of the BC loop and the FG-loop mutation in close proximity to the BC loop.

### Crystal structures of RSs bound RosC

The crystals of RosC complexed with its substrates belonged to the *C*2 space group and were isomorphous. In each crystal structure, two monomers—A and B—were present in the asymmetric unit, and they superimposed well with rms positional differences of the main chain atoms in the range of 0.17 − 0.21 Å (Table S4). Additionally, the overall structures of RosC remained largely unchanged among these three complexes, with rmsd in the range of 0.0 − 0.33 Å (Table S4). Based on these results, monomer A in each structure is discussed below.

**Fig. 6:**
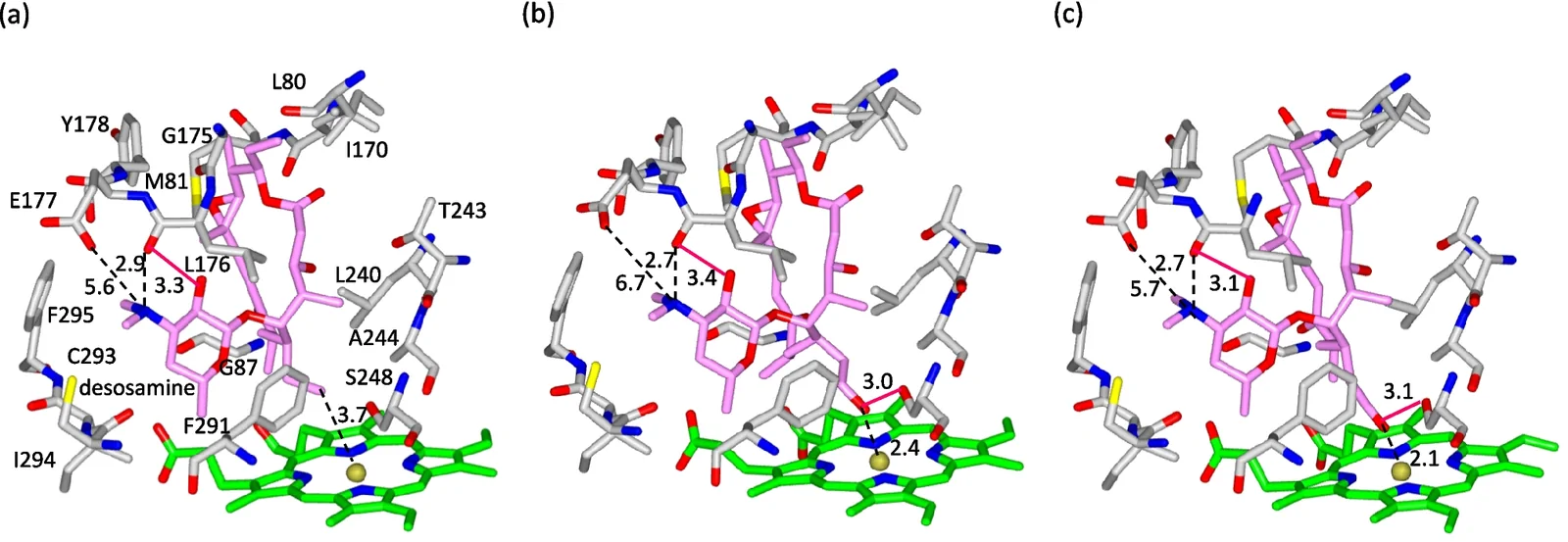
Substrate-binding pockets of RosC in complex with RS-B (**a**), RS-A (**b**), and RS (**c**). Amino acid residues within 4.0 Å from each substrate and Glu-177 are shown in *gray*. The substrates are shown in *pink*, heme is in *green*, oxygen atoms are in *red*, nitrogen atoms are in *blue*, and sulfur atoms are shown in *yellow*. Hydrogen bonds are highlighted in *magenta*. The distances are in Angstroms

The crystal structure of RosC bound to RS-B, the first substrate of RosC in RS biosynthesis, was determined at a 2.3 Å resolution (Table S2 and Fig. S3a). Similar to other P450s, RosC underwent a conformational change upon substrate binding, with the FG loop shifting to push the substrate toward the active site (Fig. S4) (Li and Poulos 1997; Sherman et al. 2006; Savino et al. 2009). RS-B was accommodated within the substrate-binding cavity of RosC in a characteristic conformation, wherein the desosamine moiety was oriented perpendicular to the aglycone plane (Fig. 6a and Fig. S5a). The carbonyl group of Leu-176 on the FG loop formed a hydrogen bond with the C2' hydroxyl group of desosamine sugar. The carbonyl group of Leu-176 is also likely to participate in electrostatic interactions with the C3' dimethylamino group of the desosamine, which exists primarily in its protonated form at neutral pH (Sherman et al. 2006). Additionally, the carboxyl group of Glu-177 was located 5.6 Å away from the protonated tertiary amine, potentially compensating for the partial positive charge. Phe-291, Cys-293, Ile-294, and Phe-295 were located within 4.0 Å of the desosamine moiety, which fit into the hydrophobic pocket formed by these residues. The macrolactone ring of RS-B was bound via hydrophobic and polar interactions with Leu-80, Met-81, and Gly-87 on the BC loop; Ile-170 and Tyr-178 on the FG loop; and Leu-240, Thr-243, Ala-244, and Ser-248 on the I helix. The hydroxylation site of RS-B at position C-20 was oriented toward the heme iron and was within 3.75 Å of it.

The crystal structures of the RosC in complex with RS-A and RS, the second and third substrates of RosC in RS biosynthesis, were both determined at a resolution of 2.3 Å (Table S2 and Fig. S3b, c). The overall binding mode was almost identical to that of RS-B. The distance between the carbonyl group of Leu-176 and the C2' hydroxyl group of the desosamine, which is involved in hydrogen bonding, was comparable (Fig. 6b, c and Fig. S5b, c). Similarly, the distance between the carbonyl group of Leu-176 and the C3' dimethylamino group of the desosamine, which is involved in electrostatic interaction, showed no significant difference. Moreover, the hydroxyl and aldehyde groups at C-20, the modification sites of RS-A and RS, each formed a hydrogen bond with Ser-248. These groups were oriented toward the heme iron, positioned within 2.4 Å and 2.1 Å of it, respectively.

## Discussion

RosC catalyzes a three-step oxidation reaction at C-20 during RS biosynthesis. The P107S/L176Q mutant of RosC is significantly less efficient in catalyzing the second and third oxidation steps in whole-cell assays using *E. coli* (Iizaka et al. 2020). In the present study, in vitro assays using purified RosC and the P107S/L176Q mutant exhibited an activity pattern consistent with that observed in the whole-cell assays. Thus, we compared the reaction time courses of RosC and the P107S/L176Q mutant with those of the first substrate, RS-B, and found that the rate of RS-A formation, which occurs during the first hydroxylation step, was markedly reduced in the mutant. Our previous study demonstrated that introducing the P107S/L176Q mutant gene into the *rosC*-deficient strain of *M. rosaria* resulted in significant accumulation of RS-A. This finding is consistent with the present results, since the markedly reduced efficiency of the mutant in the first hydroxylation step renders it the rate-limiting step in the biosynthetic pathway. Consequently, the conversion of the upstream substrate RS-B is hindered, and its immediate product, RS-A, accumulates within the cell due to inefficient conversion to subsequent products. Furthermore, the dissociation constants of RosC and the P107S/L176Q mutant for each substrate were compared using a substrate-binding assay, and no differences were observed in their affinities for RS-B. However, the mutant showed a decreased affinity for RS-A and RS, which could be one of the reasons for the reduced catalytic activity after the second step. RosC and the mutant exhibited Type II spectral changes caused by the coordination of nitrogen to the heme iron upon binding to each substrate. Generally, the binding of compounds such as imidazole, which inhibits the catalytic activity of P450, causes Type II spectral changes (Locuson et al. 2007). CYP3A4, the major human drug-metabolizing enzyme, catalyzes the hydroxylation of quiniline-4-carboxamide analogs, although its binding induces Type II spectral changes (Pearson et al. 2011). This mechanism has been proposed to involve the reorientation of the substrate into a nitrogen-coordinated form toward the heme iron and the oxidative modification site-facing form toward the heme iron. In the cocrystal structure of RosC with RS-B, a water ligand was coordinated to the heme iron at a position closer than the modification site at the C-20 position (Fig. S5a). Although nitrogen coordination to heme iron via water molecules could potentially contribute to Type II-like spectral shifts, no interaction between a water ligand coordinated to the heme and nitrogen of RS-B was observed (Lockart et al. 2018). No water ligands coordinating to the heme iron were observed in the cocrystals of RS-A and RS (Fig. S5b, c). Furthermore, for all substrates, only the orientation with the modification site at the C-20 directed toward the heme iron was observed. These trends were consistent across all subunits in the cocrystal structures with RosC. The reason RosC exhibits Type II spectral changes upon substrate binding remains unclear.

To further clarify the differences between the catalytic activities of RosC and the P107S/L176Q mutant, we determined their crystal structures. In this study, N-terminally truncated RosC and its mutant were used for crystallization, whereas full-length RosC and its mutant with an N-terminal His-tag were employed for functional assays. Although the possibility that these modifications might slightly affect enzyme properties cannot be completely excluded, the consistency of the results suggests that their influence is negligible. A comparison of the crystal structures of RosC and its mutants revealed that both mutations, Pro-107 to Ser and Leu-176 to Gln, significantly increased the structural fluctuations. The Leu-176 to Gln mutation led to pronounced conformational fluctuations in the FG and BC loops, which are important for substrate retention (Podust and Sherman 2012; DeMars et al. 2019). In the crystal structures of RosC bound to each substrate, Leu-176 was found to play a pivotal role in substrate recognition through interactions with the C-2’ hydroxy group and the C-3’ dimethylamino group of the desosamine moiety. The loss of these interactions due to the mutation of Leu-176 to Gln is considered to be one of the key factors contributing to the markedly reduced reaction rate of the P107S/L176Q mutant during the in vitro reaction with RS-B and the absence of oxidation reactions after the second and subsequent steps. Although the L176Q mutant exhibited reduced conversion efficiency in previous whole-cell assays, this reduction was not as pronounced as that of the P107S/L176Q mutant (Iizaka et al. 2020). Pro-107 appears to be distant from the active site, yet it is located on the C-helix at the base of the BC loop (Fig. 5a). The substitution to Ser is presumed to increase flexibility, thereby enhancing fluctuations of the BC loop (Fig. 5b). Thus, the Pro-107 to Ser mutation may have a synergistic effect on the decrease in catalytic activity, although no major changes in the crystal structure were observed. The substrate recognition mechanism of desosamine-mediated P450s has been elucidated through conformational analyses of the P450 PikC, which is involved in picromycin biosynthesis, and the P450 MycCI, which participates in mycinamicin biosynthesis (Sherman et al. 2006; Li et al. 2009; DeMars et al. 2016, 2019). PikC recognizes two macrolides with 12- and 14-membered rings as substrates for picromycin biosynthesis. Glu-94 and Glu-85 form salt bridges with the C-3’ dimethylamino group of desosamine when recognizing the 12- and 14-membered rings as substrates, respectively. Substitution of Glu-94 and Glu-85 with Gln resulted in a nearly complete loss of conversion of the corresponding substrate, indicating that these salt bridges are essential for desosamine anchoring. In MycCI, Ala-164 and Ser-172 form potential polar contacts with the hydroxyl group at C-2’ of desosamine, while Ser-172 also interacts with the dimethylamino group at C-3’, thereby immobilizing desosamine. Similarly, Leu-176 of RosC forms potential polar contacts with both the C-2’ hydroxyl group and C-3’ dimethylamino group of desosamine. Moreover, Leu-176 of RosC and Ala-164 of MycCI engaged in hydrogen bonding with desosamine via their backbone carbonyl groups rather than through their side chains, suggesting that the desosamine fixation patterns in RosC and MycCI are highly similar.

In the present study, co-crystallization of the P107S/L176Q mutant with each substrate was not achieved under any of the conditions. These results suggest that mutation-induced structural fluctuations in the BC and FG loops, which cover the substrate from above, hinder substrate retention. In contrast, RosC formed a cocrystal structure with each substrate, all of which bound in a nearly congruent manner. The vicinity of the P450 active center is typically hydrophobic, which may reduce the affinity of the hydroxylated site formed in the initial reaction for the active center, depending on the substrate and P450s (Seifert & Pleiss 2009; Shoji et al. 2013). In RosC, Ser-248 is hydrogen-bonded to the hydroxyl group generated in the first reaction and to the aldehyde moiety produced in the secondary reaction. This interaction is believed to support the orientation of the modification site toward the heme in the active center. Additionally, RosC binds to desosamine on each substrate, undergoing a three-step oxidative reaction through interactions with the FG loop, primarily Leu-176, as well as the hydrophobic pocket formed by Phe-291, Cys-293, Ile-294, and Phe-295. These factors may allow all three substrates to enter RosC in the same form, thereby enabling three iterative oxidative modifications at the same substrate site.

Analysis of the crystal structures of RosC and its mutants with varying catalytic efficiencies is expected to aid in protein engineering to extend the function of P450s. This could enable P450s that typically catalyze a single reaction to perform multistep oxidation reactions or allow P450s involved in multistep oxidation to selectively catalyze only the desired reaction. Such efforts will contribute to the development of new compounds and the efficient production of valuable substances. In future studies, complementary computational analyses, including substrate docking and simulations integrating quantum mechanics with molecular mechanics, could provide additional insights into substrate orientation and the electronic environment of the heme active site, further supporting protein engineering efforts.

## Supplementary Information

Below is the link to the electronic supplementary material.

Supplementary file1 (PDF 943 KB)

## Data Availability

Crystallographic data for structures reported in this article were deposited to the Protein Data Bank (PDB codes 9VGM, 9VGN, 9VGQ, 9VGP, and 9VGO for RosC, RosCP107S/L176Q, RosC complexed with RS-B, RosC complexed with RS-A, RosC complexed with RS, respectively). All other relevant data generated and analyzed in this study are available within the paper and its supplementary information.
