# Supplementary material for: Enzyme engineering of cytochrome P450 RosC provides mechanistic insights into factors controlling iterative oxidation
Source: Appl Microbiol Biotechnol. 2025 Dec 9;109(1):264. doi: 10.1007/s00253-025-13648-2 (PMC12696090; doi:10.1007/s00253-025-13648-2)
Supplement: Supplementary file 1 — Supplementary file1 (PDF 943 KB) [file 253_2025_13648_MOESM1_ESM.pdf]

Applied Microbiology and Biotechnology

Electronic Supplementary Material for:

Enzyme engineering of cytochrome P450 RosC provides mechanistic insights into factors controlling iterative oxidation

Yohei Iizaka · Hironori Suzuki · Nanako Sasa · Kihika Ishiuchi · Yuta Kumakiri · Haruki Kawasaki · Hayato Sato · Kanon Fujimoto · Shuji Noguchi · Yojiro Anzai

Faculty of Pharmaceutical Sciences, Toho University, 2-2-1 Miyama, Funabashi, Chiba, Japan

Correspondence:
Yohei Iizaka
Faculty of Pharmaceutical Sciences, Toho University, 2-2-1 Miyama, Funabashi, Chiba 274-8510, Japan.
Tel.: +81 47 472 2068
Fax.: +81 47 472 2086
E-mail: yohei.iizaka@phar.toho-u.ac.jp

Table S1

PCR primers used in this study.

Primer	Purpose	Sequence (5'-3')
rosC_Nd-F	Construction of plasmid expressing RosC	GCATATGACGCAGACGGACAACGCA
rosC_Xh-R	Construction of plasmid expressing RosC	TTCTCGAGTCAGCCGCGGACCACCTGGA
rosC_ND21-F	Construction of plasmid expressing RosC or $RosC_{P107S/L176Q}$ lacking the N-terminal 21 residues	GCATATGGGGCTGCTGGACTGGTTCGC

Table S2 Data collection and refinement statistics.

Protein	RosC	$RosC_{P107S/L176Q}$
Ligand	No ligand	No ligand
PDB ID	9VGM	9VGN
Data collection		
X-ray source	PF BL17A	PF BL5A
Wavelength (Å)	0.9800	1.0000
Space group	$C222_1$	$C222_1$
Cell dimensions		
a, *b*, *c* (Å)	102.9, 169.0, 210.7	102.2, 167.8, 211.4
α, *β*, *γ* (°)	90, 90, 90	90, 90, 90
Resolution (Å) (outer shell)	48.1–2.15 (2.28–2.15)	49.1–2.45 (2.60–2.45)
No. of obs. Reflections	1,360,991 (215,677)	440,753 (71,383)
No. of unique reflections	192,889 (31,017)	128,462 (20,658)
Redundancy	7.1 (7.0)	3.4 (3.5)
Completeness (%)	99.9 (99.5)	99.6 (99.4)
$I/\sigma(I)$	14.00 (1.94)	12.25 (1.50)
$R_{sym}(I)$ (%)	9.9 (115.2)*	7.0 (71.1)
$CC_{1/2}$	99.9 (66.4)	99.8 (71.6)
Refinement		
R_{work}/R_{free} (%)	18.1/21.8	21.0/26.7
RMSD		
Bond length (Å)	0.007	0.008
Bond angles (°)	0.979	1.033
No. of mol. in ASU	3	3
B-factors (Å^2)	45.73	63.30
Ramachandran plots		
Favored (%)	98.26	95.39
Allowed (%)	1.74	4.61
Outlier (%)	0	0

Protein	RosC	RosC	RosC
Ligand	RS-B	RS-A	RS
PDB ID	9VGQ	9VGP	9VGO
Data collection			
X-ray source	PF-AR NE3A	PF BL5A	PF BL5A
Wavelength (Å)	1.0000	1.0000	1.0000
Space group	*C*2	*C*2	*C*2
Cell dimensions			
a, *b*, *c* (Å)	128.0, 96.0, 96.3	124.8, 95.3, 95.7	127.7, 95.7, 95.9
α, *β*, *γ* (°)	90, 108.9, 90	90, 106.6, 90	90, 109.1, 90
Resolution (Å) (outer shell)	48.1–2.30 (2.44–2.30)	48.1–2.30 (2.44–2.30)	48.1–2.30 (2.44–2.30)
No. of obs. reflections	163,839 (26,903)	160,515 (24,737)	165,017 (26,378)
No. of unique reflections	91,328 (14,919)	88,633 (13,970)	91,684 (14,714)
Redundancy	1.8 (1.8)	1.8 (1.8)	1.8 (1.8)
Completeness (%)	94.7 (96.1)	94.3 (91.7)	95.9 (95.1)
$I/\sigma(I)$	7.88 (1.71)	7.53 (1.54)	7.72 (1.26)
$R_{sym}(I)$ (%)	9.2 (81.0)	7.8 (54.8)	8.4 (60.5)
$CC_{1/2}$	99.4 (55.3)	99.7 (76.9)	99.5 (68.8)
Refinement			
R_{work}/R_{free} (%)	19.2/25.5	21.7/26.3	19.8/25.5
RMSD			
Bond length (Å)	0.009	0.007	0.008
Bond angles (°)	1.080	0.995	0.992
No. of mol. in ASU	2	2	2
B-factors (Å^2)	47.12	43.97	43.23
Ramachandran plots			
Favored (%)	96.1	96.9	96.6
Allowed (%)	3.9	3.1	3.4
Outlier (%)	0	0	0

Values in parentheses are for the highest resolution shell.

*The R_{sym} value in the highest-resolution shell is high, but the data quality was sufficient for reliable structure determination, as indicated by reasonable $CC_{1/2}$ and completeness.

Table S3

Detection of RSs concentrations during the time course of enzymatic reactions catalyzed by NHis-RosC and NHis-RosC$_{P107S/L176Q}$.

RosC	time (min)	Concentrations (μM)			
		RS-B	RS-A	RS	RS-F
NHis-RosC	0	93.0 ± 5.9	0.0	0.0	0.0
	5	71.0 ± 6.5	22.1 ± 8.0	0.0	0.0
	30	37.4 ± 8.7	58.6 ± 8.6	2.0 ± 0.1	0.0
	60	19.8 ± 8.4	76.0 ± 4.8	7.9 ± 2.2	0.0
	120	2.8 ± 3.4	71.5 ± 8.0	27.3 ± 12.5	0.0
	180	0.0	43.7 ± 0.5	54.8 ± 3.1	0.0
	360	0.0	11.6 ±9.7	82.2 ± 2.9	5.3 ± 7.5
	540	0.0	0.0	85.8 ± 7.6	13.5 ± 4.3
NHis-RosC$_{P107S/L176Q}$	0	97.8 ± 1.2	0.0	0.0	0.0
	5	101.7 ± 3.5	0.0	0.0	0.0
	30	88.9 ± 5.4	7.9 ± 2.2	0.0	0.0
	60	81.0 ± 5.4	14.4 ± 4.3	0.0	0.0
	120	75.7 ± 0.6	20.5 ± 2.2	0.0	0.0
	180	70.4 ± 4.1	30.0 ± 1.5	0.0	0.0
	360	66.3 ± 2.7	35.6 ± 1.9	0.0	0.0
	540	60.5 ± 8.4	36.5 ± 6.9	0.0	0.0

The average concentrations and their standard deviations reflect the results from duplicate experiments.

Table S4

Detailed list of rmsd values (Å) between monomers for each crystal structure.

		RosC			$RosC_{P107S/L176Q}$			RosC RS-B		RosC RS-A		RosC RS	
		A	B	C	A	B	C	A	B	A	B	A	B
RosC	A		0.218	0.293	0.312	0.434	0.381	1.443	1.305	1.425	1.437	1.465	1.471
	B			0.270	0.286	0.450	0.436	1.449	1.377	1.485	1.461	1.472	1.501
	C				0.356	0.340	0.485	1.447	1.462	1.469	1.480	1.472	1.495
$RosC_{P107S/L176Q}$	A					0.380	0.368	1.397	1.407	1.409	1.389	1.410	1.435
	B						0.373	1.354	1.392	1.361	1.385	1.364	1.372
	C							1.347	1.378	1.345	1.325	1.362	1.369
RosC RS-B	A								0.213	0.301	0.325	0.197	0.245
	B									0.318	0.331	0.241	0.220
RosC RS-A	A										0.172	0.235	0.265
	B											0.270	0.279
RosC RS	A												0.175
	B												

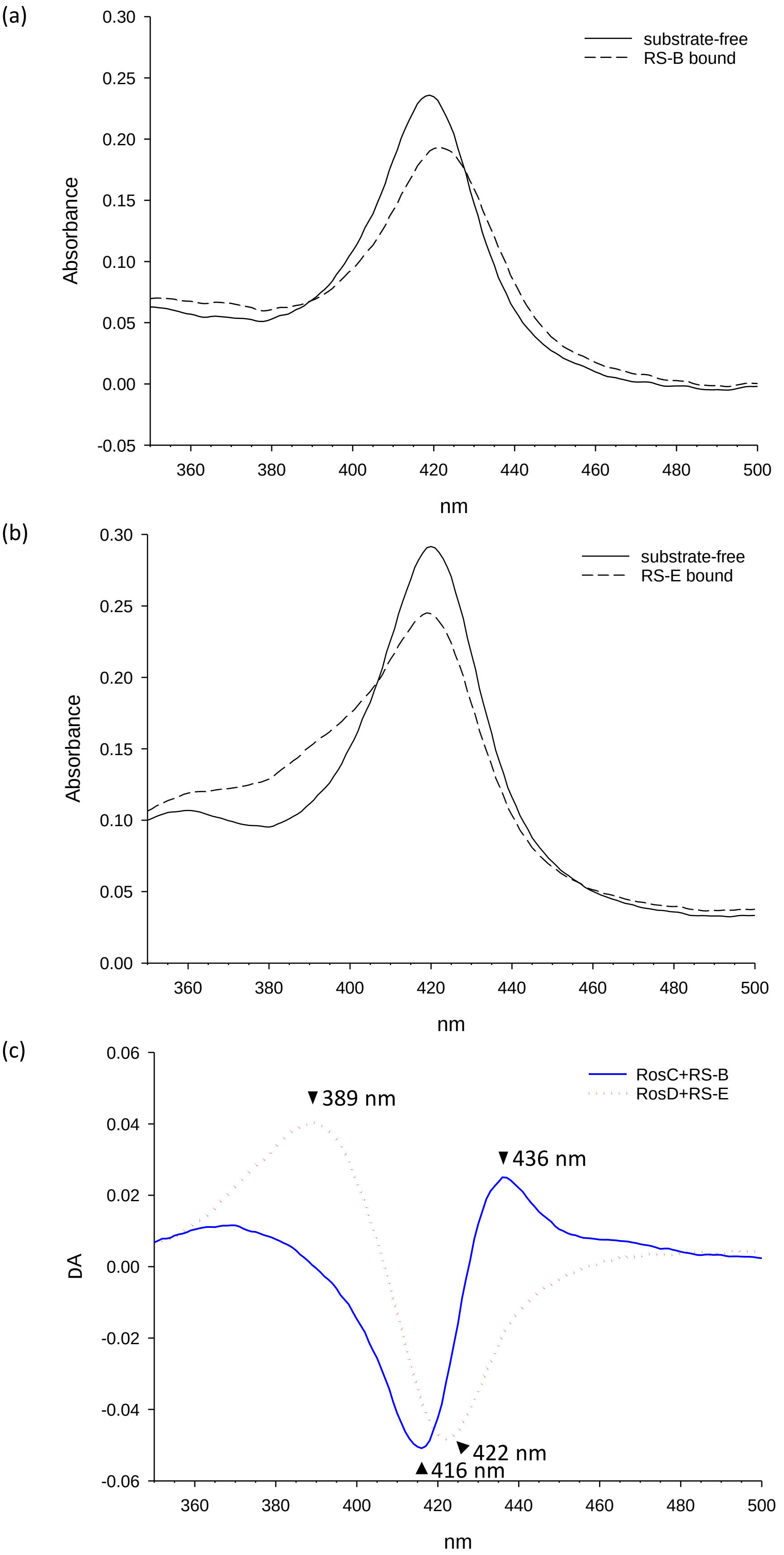

Fig. S1 UV-visible absorption and substrate-induced difference spectra of RosC with RS-B and RosD with 20-deoxo-20-dihydro-12,13-deepoxyrosamicin (RS-E). (a) Absorption spectra of 1 μM purified NHis-RosC in the absence and presence of 10 μM RS-B. (b) Absorption spectra of 1 μM purified NHis-RosD in the absence and presence of 10 μM RS-E. (c) Corresponding difference spectra showing substrate-induced spectral shifts.

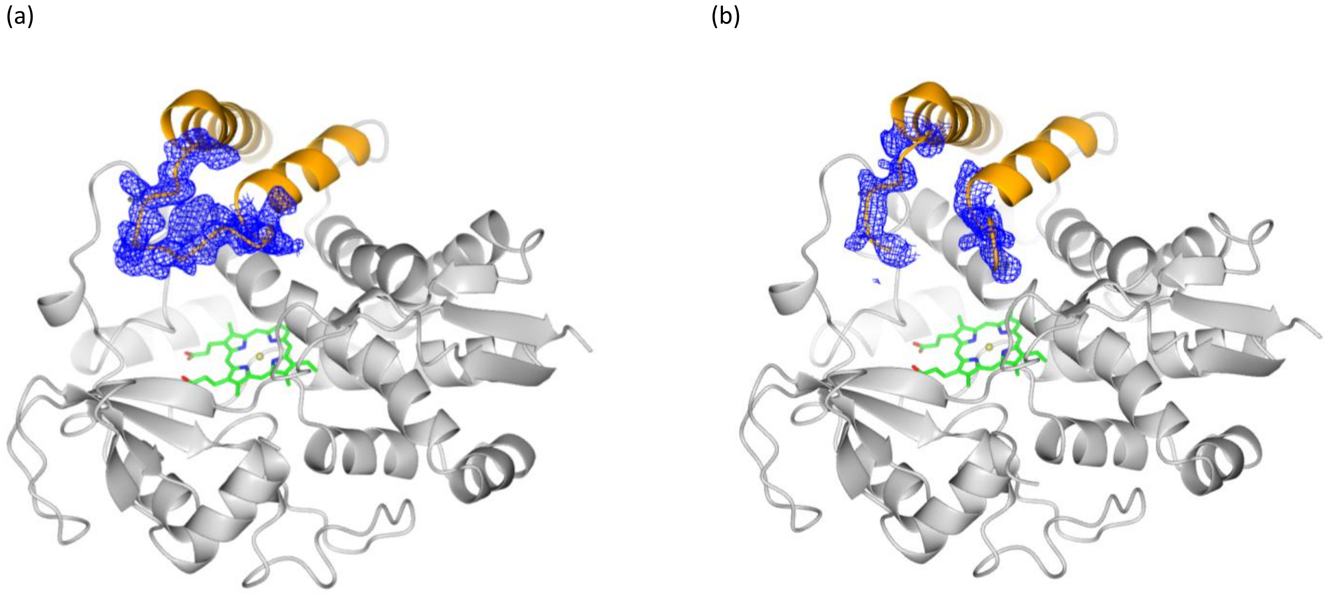

Fig. S2 Overall structures with electron density maps of RosC (a) and $RosC_{P107S/L176Q}$ (b). *Blue mesh* surrounding the FG-loop region of RosC and $RosC_{P107S/L176Q}$ represents the 2*Fo-Fc* electron density map contoured at 1.0 σ. The FG helix and loop region is shown in *orange*, and heme in *green.*

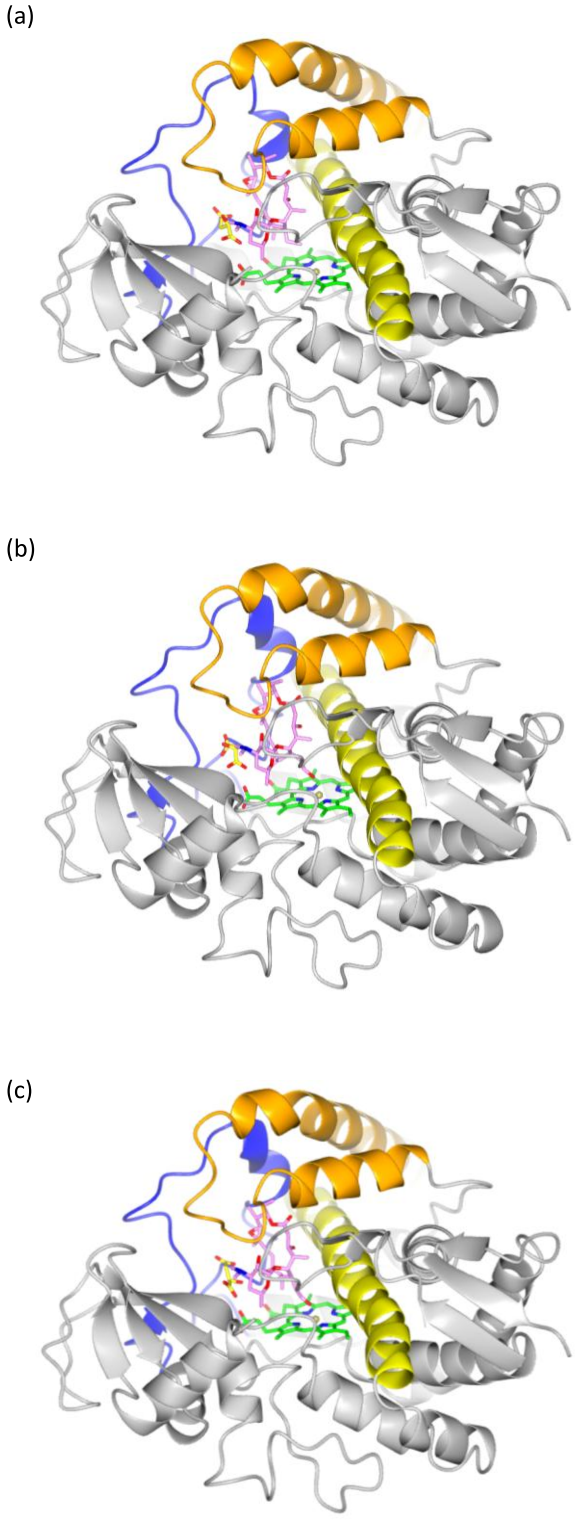

Fig. S3 Overall structure of RosC bound to RS-B (a), RS-A (b), and RS (c). The substrates, heme, and malic acid are shown as *stick models,* in *pink, green, and yellow,* respectively. Malic acid, one of the components of the crystallization reagent, was observed in the binding pocket of RosC. However, it is unlikely to affect substrate binding. The BC loop region, FG helix region, and I helix region are shown in *blue*, *orange*, and *yellow*, respectively.

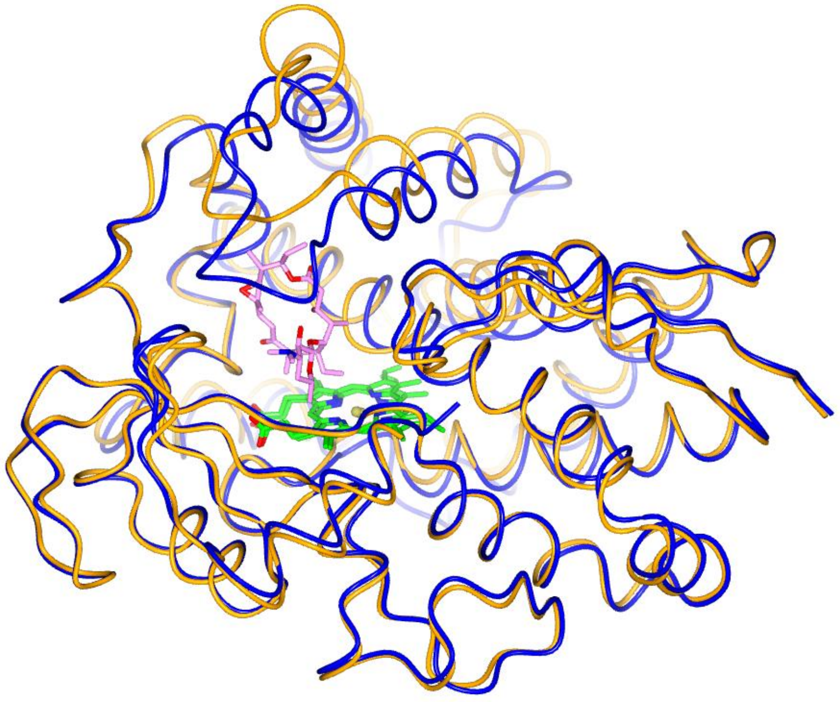

Fig. S4 Overlay of RosC structures in ligand-free state and in complex with RS-B. The ligand-free and RS-B bound forms are shown as orange and blue loop models, respectively. Hemes and RS-B are depicted as green and pink stick models, respectively.

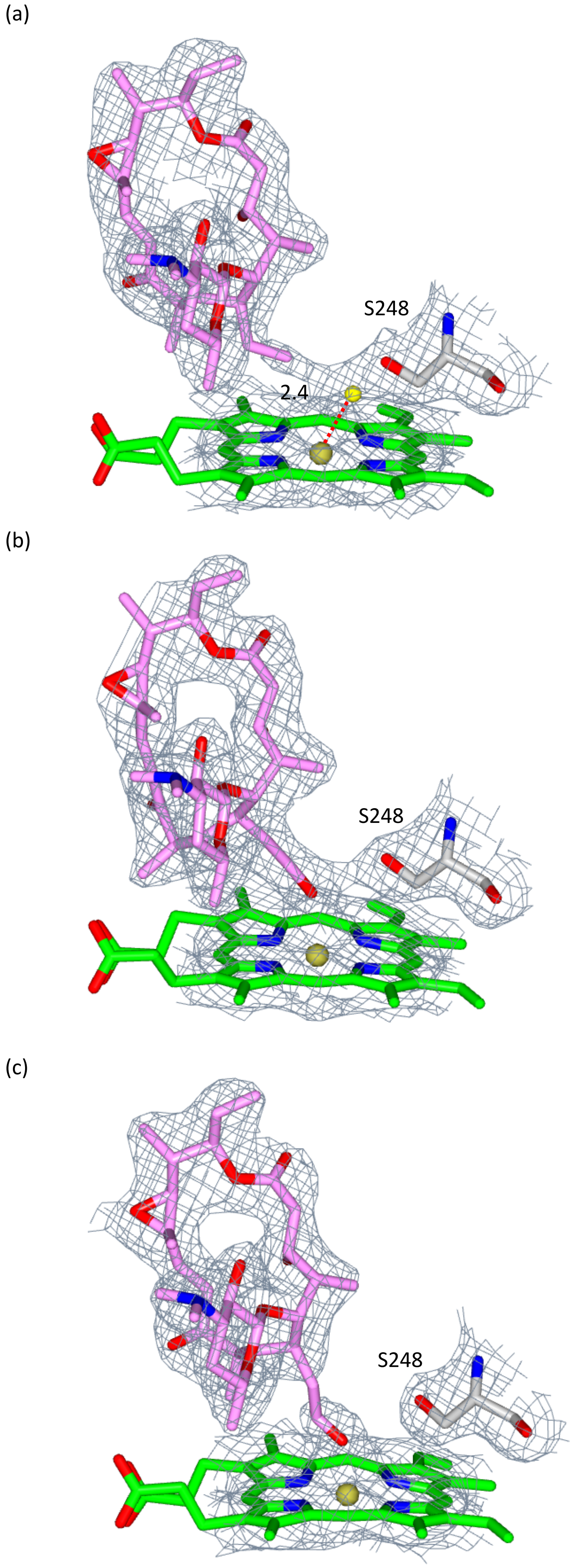

Fig. S5 Electron density maps around the active sites of RosC complexed with substrates RS-B (a), RS-A (b), and RS (c). *Gray mesh* surrounding each substrate, Ser-248, the water molecule, the heme represents the 2*Fo*-*Fc* electron density map contoured at 1.0 σ. Only the heme iron and a portion of the porphyrin ring are shown for clarity. The water molecule is shown in *yellow*, substrates in pink, heme in *green*, Ser-248 in *gray*, oxygen atoms in *red*, and nitrogen atoms in *blue*. The distances are in Angstroms.
